# Short Duration Small Sided Football and to a Lesser Extent Whole Body Vibration Exercise Induce Acute Changes in Markers of Bone Turnover

**DOI:** 10.1155/2016/3574258

**Published:** 2016-11-29

**Authors:** J. L. Bowtell, S. R. Jackman, S. Scott, L. J. Connolly, M. Mohr, G. Ermidis, R. Julian, F. Yousefian, E. W. Helge, N. R. Jørgensen, J. Fulford, K. M. Knapp, P. Krustrup

**Affiliations:** ^1^Sport and Health Sciences, College of Life and Environmental Sciences, St Luke's Campus, University of Exeter, Exeter, UK; ^2^Centre of Health and Human Performance, Department of Food, Nutrition and Sport Science, University of Gothenburg, Gothenburg, Sweden; ^3^Centre of Health Sciences, Faculty of Natural and Health Sciences, University of the Faroe Islands, Tórshavn, Faroe Islands; ^4^Department of Sports Science and Physical Education, Democritus University of Thrace, Komotini, Greece; ^5^Institute of Sports and Preventive Medicine, University of Saarland, Saarbrücken, Germany; ^6^Copenhagen Center for Team Sport and Health, Department of Nutrition, Exercise and Sports, University of Copenhagen, Copenhagen, Denmark; ^7^Research Center for Ageing and Osteoporosis, Department of Clinical Biochemistry, Copenhagen University Hospital, Copenhagen, Denmark; ^8^NIHR Exeter Clinical Research Facility, University of Exeter Medical School, Exeter, UK; ^9^University of Exeter Medical School, University of Exeter, Exeter, UK; ^10^Department of Sports Science and Clinical Biomechanics, SDU Sport and Health Sciences Cluster (SHSC), University of Southern Denmark, 5230 Odense, Denmark

## Abstract

We aimed to study whether short-duration vibration exercise or football sessions of two different durations acutely changed plasma markers of bone turnover and muscle strain. Inactive premenopausal women (*n* = 56) were randomized to complete a single bout of short (FG15) or long duration (FG60) small sided football or low magnitude whole body vibration training (VIB). Procollagen type 1 amino-terminal propeptide (P1NP) was increased during exercise for FG15 (51.6 ± 23.0 to 56.5 ± 22.5 *μ*g·L^−1^, mean ± SD, *P* < 0.05) and FG60 (42.6 ± 11.8 to 50.2 ± 12.8 *μ*g·L^−1^, *P* < 0.05) but not for VIB (38.8 ± 15.1 to 36.6 ± 14.7 *μ*g·L^−1^, *P* > 0.05). An increase in osteocalcin was observed 48 h after exercise (*P* < 0.05), which did not differ between exercise groups. C-terminal telopeptide of type 1 collagen was not affected by exercise. Blood lactate concentration increased during exercise for FG15 (0.6 ± 0.2 to 3.4 ± 1.2 mM) and FG60 (0.6 ± 0.2 to 3.3 ± 2.0 mM), but not for VIB (0.6 ± 0.2 to 0.8 ± 0.4 mM) (*P* < 0.05). Plasma creatine kinase increased by 55 ± 63% and 137 ± 119% 48 h after FG15 and FG60 (*P* < 0.05), but not after VIB (26 ± 54%, NS). In contrast to the minor elevation in osteocalcin in response to a single session of vibration exercise, both short and longer durations of small sided football acutely increased plasma P1NP, osteocalcin, and creatine kinase. This may contribute to favorable effects of chronic training on musculoskeletal health.

## 1. Introduction

Osteoporosis is characterized by low bone mass and microarchitectural deterioration, resulting in increased susceptibility to fracture. Bone mineral density (BMD) decreases with age particularly amongst women, with a sharp decline following the menopause, such that 1 in 2 women over the age of 50 y in the UK experiences a fracture in the remaining lifespan [1]. With the ageing population demographic, annual fracture incidence in postmenopausal women in the UK is expected to increase by 17% from 2010 to reach 262,847 fractures by 2020 [2]. Physical activity during childhood, adolescence, and young adulthood maximizes peak bone mass, whereas physical activity during adulthood decreases rate of bone loss [3] both of which will diminish future fracture risk.

Mechanical loads imposed upon bone through gravitational loading and internal loading through muscle contraction stimulate osteogenesis [4] although the specific mechanisms involved are not yet fully understood. However animal studies suggest that bone formation is positively correlated with peak strain magnitude and rates, once a threshold level is exceeded [5, 6]. This is corroborated in humans, whereby physical activities that generate high accelerations, ground reaction forces, and power output seem to provide the most effective osteogenic response, whether evaluated by long-term changes in BMD [7] or by short-term changes in biochemical markers of bone resorption and formation [8, 9].

In recent publications, we have demonstrated that football training is associated with strong osteogenic effects in women. Tibial BMD increased by 2-3% in untrained premenopausal women after 14-15 weeks football training [10]. Moreover, after 15 weeks football training sedentary middle aged hypertensive women experienced significant increases in plasma markers of bone formation, and increased leg bone mass, compared with controls [11]. Football participation seems to provide an unusual osteogenic stimulus, since characteristic football actions such as rapid sprints, stops, jumps, and turns, performed with intermittence, generate high rates of mechanical loading and strain distributions that are different from habitual physical activity. Such stimulus characteristics have been identified as optimal in both* in vitro* [5] and* in vivo* [6] studies of bone loading. In support of this notion, a recent study [12] has reported a significant correlation between the increase in leg bone mass, and the number of individual accelerations, decelerations, and the volume of high-intensity running performed during small sided football training with elderly men with prostate cancer (*r* = 0.59–0.65). This association between specific high-intensity movements performed during football and changes in bone mass supports the hypothesis that the dynamic actions of football, and more specifically, their rate and frequency of occurrence underlie the osteogenic adaptations in bone [13]. However, the minimum duration, intensity, and frequency that may be required to influence bone adaptation and metabolism are still largely unknown.

There has also been interest in the osteogenic potential of whole body vibration (VIB) which provides passive mechanical loading to the skeleton, since the oscillations generated by the vibrating platform impart accelerations to the body [14]. Such training may be more acceptable in individuals either unable or unwilling to perform high impact exercise. In contrast to small sided football, VIB does not require large increases in energy expenditure and hence metabolic and cardiovascular demands are limited [15, 16]. Animal studies suggest that chronic VIB training increases BMD and bone strength [17] and reduces the concentration of bone resorption markers [17]. A recent meta-analysis found that chronic VIB resulted in statistically significant, but clinically small improvements in BMD in postmenopausal women, moderate effects in children and adolescents, but no effect on BMD in young adults [18]. Low magnitude side alternating vibration (6 × 1 min bouts at 12.6 Hz and 3 mm amplitude, three times per week), such as that employed in the present study, has also been shown to increase bone mineral density in postmenopausal women after 8 months of vibration training [19]. These favorable adaptations in BMD must presumably occur as a consequence of bone formation exceeding the rate of bone resorption resulting in net gain of bone mineral. Chronic VIB training has been shown to either decrease C-terminal telopeptide of type 1 collagen (CTX-1), a marker of bone resorption in both postmenopausal women (vertical vibration 12 Hz and 0.5 mm, 0.3 g; [20]) and young men (side alternating vibration 18 Hz and 4 mm, 2.6 g, for 4 weeks increasing to 22 Hz and 4 mm, 3.8 g, for last four weeks [21]), or to increase N-terminal propeptide type I procollagen (P1NP), a marker of bone formation in elderly adults (side alternating vibration at 29.8 Hz 2.9 mm, 3.6 g, [22]). However, these chronic studies cannot distinguish whether these effects relate to a chronic change in bone turnover or accrual of a series of acute responses after each training session.

To our knowledge only two studies have examined the acute effects of VIB (20 Hz, 3.38 mm, side alternating vibration) on bone turnover markers up to 30 min postexercise. In both recreationally active young men [23] and women (20–30 y) [24], CTX-1 was reduced to a greater extent 30 min after exercise when VIB was performed prior to resistance exercise, compared to resistance exercise alone. However, data for either acute exposure to VIB alone or acute osteogenic responses to short or longer duration small sided football have not previously been reported. Thus, the purpose of the present study was to determine whether a single bout of short or long duration small sided football or low magnitude side alternating whole body vibration training would induce favorable responses in markers of bone turnover and muscle strain in premenopausal inactive women.

## 2. Methods

### 2.1. Participants

Fifty-six participants with a mean age of 38.1 ± 5.4 years (range: 26–46 years) participated in the study and were recruited via poster and newspaper advertisements. All of the participants were nonsmokers, were not pregnant or taking any medication known to affect bone metabolism, and had not been involved in any regular exercise training for at least two years. All participants had little or no experience of playing football and were premenopausal and menstruating regularly. Habitual calcium intake was estimated using the NIH short calcium questionnaire (SCQ-2002). All participants gave their written informed consent and the study was approved by the National Research Ethics Service (NRES) 12/SW/0045 and the institutional research ethics committee (NHS 2012/329).

### 2.2. Experimental Design

Participants were randomly assigned to three groups, which participated in short duration (13.5 min small sided football, FG15, *n* = 18) or longer duration (4 × 13.5 min small sided football and 1.5 min recovery, FG60, *n* = 18) or whole body vibration (13.5 min, VIB, *n* = 20). Participant characteristics are presented in Table 1. To examine the association between exercise intensity during football and acute metabolic responses, the acute stimulus provided by FG15 and FG60 was quantified using GPS and HR data.

### 2.3. Experimental Protocol

Participants arrived at the laboratory after an overnight fast (from midnight), and all testing commenced between 8 and 8:30 am and no food was consumed until at least 30 min after exercise. A cannula was inserted into an antecubital vein for serial blood sampling immediately before, during (after the first 13.5 bout in FG60 only), and after the training session (immediately, 30 min and 48 h postexercise). The football sessions took place outdoor on natural grass on 15–25 m wide and 20–35 m long pitches, with 65–80 m^2^ per player as used in previous studies for untrained women [25]. The ambient temperature was 8–14°C. Sessions were organised as small sided games (2v2, 3v3, and 4v4) using 1.2 × 5 m goals and alternating goalkeepers, which have been shown to be an intense and versatile training type for untrained women with many fast runs, sideways, and backwards runs along with many specific intense actions such as dribbles, shots, and turns [25, 26]. The football sessions commenced with a standardised warm-up consisting of 1.5 min of intermittent low-intensity running, that is, the first six 20-m shuttles of the Yo-Yo intermittent endurance level 1 test (YYIE1, [27]). Each 40-metre run was followed by a 5 s active recovery during which participants walked 2.5 m twice. The sessions were supervised by university staff but the players acted as referees themselves. For FG60, the one-hour football session was split into 4 × 13.5 min bouts followed by 1.5 min recovery, and FG15 completed one block of exercise (1 × 13.5 min session).

Whole body vibration training was completed on a side alternating platform (Galileo, Novotec Medical, Pforzheim, Germany) under University of Exeter staff supervision. Participants completed a 3 min warm-up standing on the platform with slightly bent knees during vibration at 6 Hz and 3 mm amplitude (0.2 G). Participants then completed a sequence of seven exercises whilst standing on the platform: static squat, dynamic squat, pelvic tilt, back extension and flexion, static squat, dynamic squat, and pelvic tilt each lasting 1 min whilst being exposed to vibration at 12 Hz and 3 mm peak to peak amplitude (0.9 G). Each exercise was separated by 1 min standing recovery. Participants wore only socks during the vibration and were allowed to rest their hands on a support bar in order to maintain balance, if required. The exercises were selected to maximize vibration transmission through the pelvis and hips, sites that are particularly vulnerable to bone mineral loss due to their high trabecular bone content.

### 2.4. Data Collection

Participants were familiarised with all testing procedures on at least one occasion before the start of the study. Participants were asked not to perform any physical activity for 48 hours prior to the test day. Participants completed a questionnaire prior to their scans which included any clinical risk factors for osteoporosis in addition to their current and previous menstruation histories and contraceptive use.

### 2.5. Body Composition

Body fat percentage, lean body mass, and bone mineral density were determined by DXA scanning (GE Lunar Prodigy, GE Healthcare, Bedford, UK) prior to the test day.

### 2.6. Blood Analysis

At each time point venous blood samples were aspirated to sodium fluoride, serum separator, EDTA, and lithium heparin vacutainers. For technical reasons, blood data for all time points are only available for 41 participants (FG60: *n* = 11; FG15, *n* = 13; VIB, *n* = 17). Whole blood was analysed for lactate and glucose (YSI 2300 analyser, Yellow Spring Instrument, OH, US), and the remaining vacutainers were centrifuged at 4000 rpm at 4°C with the resultant plasma stored at −80°C for subsequent analysis.

Plasma CTX-I was measured using the IDS-iSYS CTX-I (CrossLaps®) (Immunodiagnostic Systems, plc, Tyne and Wear, UK) assay. Plasma P1NP was measured by the IDS-iSYS intact PINP assay (Immunodiagnostic Systems). Plasma osteocalcin was analyzed using the N-Mid Osteocalcin assay (Immunodiagnostic Systems). All assays were carried out on the dedicated iSYS automated analyzer according to the manufacturer's instructions. All three assays are chemiluminescence immunoassays.

For each assay the sample aliquots were kept frozen and kept at minus 80 degrees until the day of analysis. All samples were analyzed using one single batch of each assay. Assay performance was verified using the manufacturers' control specimens and derived in our lab. All three analytes are accredited according to the DS/EN ISO 15189:2013 standard. The intermediary precision expressed as coefficient of variation for CTX-I was 5.3% (at CTX-I concentration 0.213 *μ*g/L) and 3.4% (0.869 *μ*g/L), and 3.5% (2.113 *μ*g/L). For P1NP the intermediary precision was 5.4% (18.96 *μ*g/L), 6.5% (48.48 *μ*g/L), and 6.1% (122.10 *μ*g/L). Finally, for osteocalcin the intermediary precision was 3.0% (8.73 *μ*g/L), 3.6% (27.58 *μ*g/L), and 3.5% (68.70 *μ*g/L). The lower limits of quantitation for the three parameters were 0.03 *μ*g/L for CTX-I, 2.0 *μ*g/L for P1NP, and 2.0 *μ*g/L for osteocalcin. These are manufacturer derived values as the analyses are CE-marked.

Plasma ammonia concentration was measured by an enzymatic kinetic assay method (Roche Diagnostics, Mannheim, Germany) using a Hitachi 912 Automatic Analyzer (Roche Diagnostic, Mannheim, Germany). Plasma free fatty acid (FFA) concentration was measured using an enzymatic colorimetric method (Wako Chemicals, Inc., Richmond, VA) adapted for the Hitachi 912 Automatic Analyzer (Roche Diagnostics, Mannheim, Germany). Plasma CK activity was analysed by an enzymatic kinetic assay method (Roche Diagnostic, Mannheim, Germany) using a Hitachi 912 Automatic Analyzer (Roche Diagnostic).

### 2.7. Movement Analyses and Heart Rate Measurements

Participants' movement pattern and heart rate (HR) were recorded during the warm-up and the FG15 and FG60 sessions using a portable 15 Hz global positioning system (GPS; SPI Pro X, GPSports, Canberra, Australia) and a Polar T34 belt (Polar Electro Oy, Kempele, Finland). The participants' movements were divided into the following categories (as in Randers et al. [27]): standing (0–0.4 km/h), walking (0.4–5 km/h), jogging (5–7 km/h), low-speed running (7–9 km/h), moderate-speed running (9–11 km/h), high-speed running (11–15 km/h), and sprinting (>15 km/h). The speed categories were computed into the Team AMS software to provide speed zones for subsequent transfer to Microsoft Excel. Total distance, peak speed, and high intensity running distance (sum of running, high-speed running, and sprinting) were also calculated. Data were downloaded to Team AMS v. 1.5 (GPSports, Canberra, Australia) and an experienced user subsequently split the data into subsections to represent the 4 × 13.5 minute bouts of exercise. Minimum, mean and maximum heart rates, and speeds were automatically calculated by the Team AMS system. The individual maximal heart rate was determined as the highest heart rate recorded during the exercise session.

### 2.8. Statistics

All statistical analyses were performed using the Statistical Package for the Social Sciences (SPSS, v. 20, SPSS Inc, Chicago, IL, USA). Data were analysed by two-way mixed model analysis of variance (ANOVA) for time (basal, immediately after 13.5 min exercise, 30 min postexercise, and 48 h postexercise) versus condition (FG15, FG60, and VIB). Repeated measures data were checked for the assumption of Sphericity using Mauchly's test. The Greenhouse Geisser correction factor was used to adjust the degrees of freedom if Mauchly's test was significant (*P* < 0.05). Between-group data were checked for the assumption of homogeneity of variance using Levene's test. Between-group data for peak change in the bone markers were evaluated using one way ANOVA. For FG60, change from 13.5 to 54 min exercise was assessed via paired *t*-test. Data are presented as mean ± SD. A significance level of 0.05 was chosen.

## 3. Results

### 3.1. Exercise Intensity

Distances covered in each exercise intensity zone for the first 13.5 minutes of exercise were not different between FG15 and FG60 when comparing (data not shown). Nor were there differences in the distance covered with high intensity running, number of accelerations and decelerations, and total distance covered between FG15 and the first 13.5 min training session in FG60 (Table 2). However, approximately 3-fold more distance was covered in all exercise intensity zones and three times more accelerations and decelerations were completed during the entire 60 minutes of training in FG60 compared to the FG15 (*P* < 0.001, Table 2).

There were no significant differences in mean HR in FG15 and FG60 when comparing first 13.5 min of training (156 ± 14 and 160 ± 19 bpm, resp.), and average heart rate over the entire 60 min was not different to the first 13.5 min of training in FG60 (Table 2). HR was not elevated above basal during vibration training.

### 3.2. Blood Metabolites

**Figure 1:**
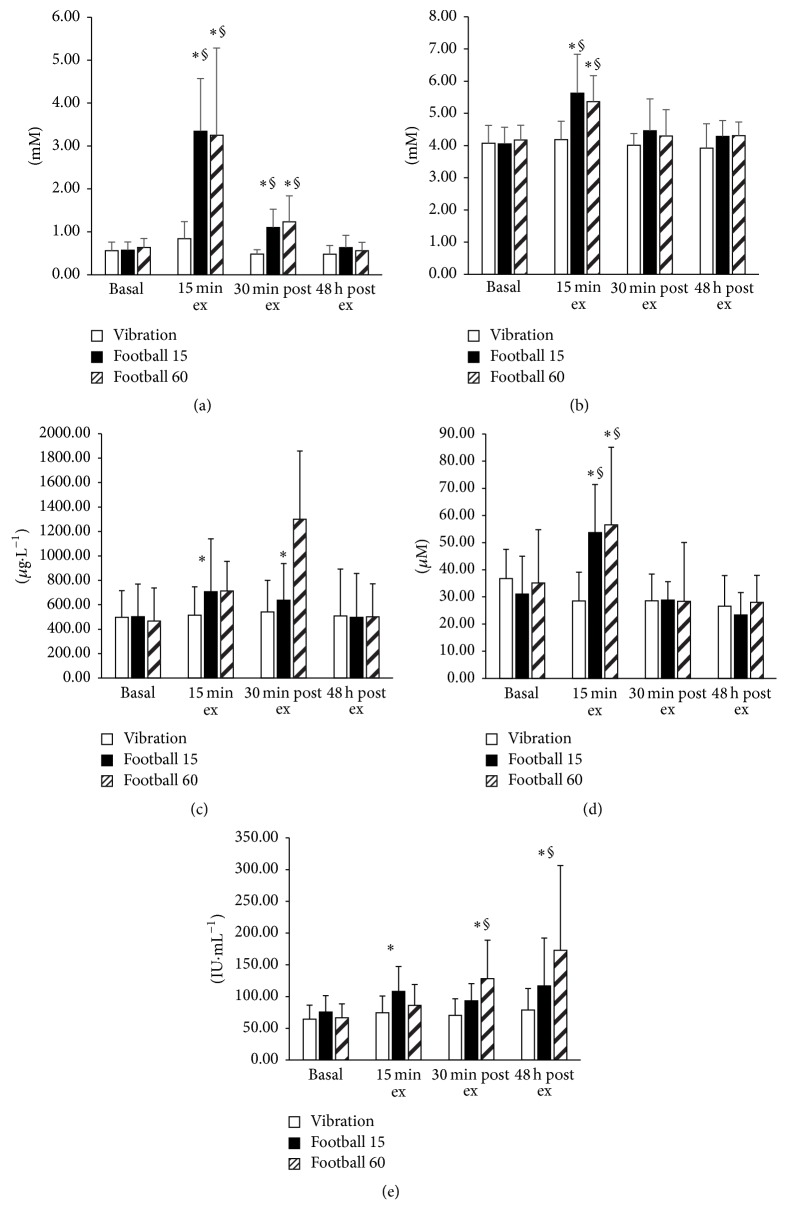
Blood lactate (a), blood glucose (b), plasma free fatty acid (c), and plasma ammonia (d) concentrations and plasma creatine kinase activity (e) before, during, and after the 15-minute (FG15, *n* = 12) and 60-minute (FG60, *n* = 6) football training session, and whole body vibration training (VIB, *n* = 7). ^*∗*^Significantly different from 0 min and ^§^significantly different from VIB at the same time point (*P* < 0.05).

There was a main effect of time (*P* < 0.001) and a condition time interaction effect (*P* = 0.001, Figure 1(a)) for blood lactate concentration, whereby after 13.5 min of exercise lactate concentration was significantly elevated relative to basal (0.6 ± 0.2 mM) in both FG15 (3.4 ± 1.2 mM) and FG60 (3.3 ± 2.0 mM) and remained elevated after 54 min of exercise in FG60 (2.6 ± 1.7 mM), and after 30 min recovery in both groups. However, there was no significant change in blood lactate concentration immediately after VIB (0.8 ± 0.4 mM).

There was a main effect of time (*P* < 0.001) and a condition by time interaction effect (*P* = 0.031, Figure 1(b)) for blood glucose concentration, whereby after 13.5 minutes of exercise glucose concentration was significantly elevated relative to basal (4.1 ± 0.5 mM) in both FG15 (5.6 ± 1.2 mM) and FG60 (5.3 ± 0.8 mM) and remained elevated after 54 minutes of exercise in FG60 (5.2 ± 1.1 mM), and after 30 min recovery in both groups. However, there was no significant change in blood glucose concentration in response to a vibration training session (4.2 ± 0.6 mM). Plasma FFA concentration was significantly elevated compared to basal values after 13.5 min exercise and 30 min after exercise (main effect of time *P* = 0.002, Figure 1(c)) but there was no condition time interaction effect. Plasma FFA concentration was significantly higher after 54 min (1267 ± 581 *μ*g·L^−1^) than 13.5 min exercise in FG60 (713 ± 243 *μ*g·L^−1^, *P* < 0.001). In similar fashion to plasma lactate, plasma ammonia concentration was significantly higher after 13.5 min exercise in both FG15 and FG60, but not affected by VIB (condition by time interaction effect: *P* < 0.001, Figure 1(d)). In contrast however, plasma ammonia concentration was lower after 54 min (56.6 ± 28.6 *μ*M) compared to 13.5 min exercise (40.1 ± 15.5 *μ*M, *P* = 0.017) in FG60.

There was a main effect of time (*P* < 0.001) and a condition by time interaction effect (*P* < 0.001, Figure 1(e)) for plasma CK activity, whereby after 13.5 minutes of exercise CK activity was significantly elevated relative to basal (69 ± 23 IU·mL^−1^) in both FG15 (108 ± 39 IU·mL^−1^) and FG60 (86 ± 33 IU·mL^−1^) and remained elevated after 54 minutes of exercise in FG60 (119 ± 32 IU·mL^−1^), and after 30 min and 48 h recovery in both groups. However, there was no significant change in CK activity in response to the vibration training session (74 ± 26 IU·mL^−1^).

### 3.3. Bone Markers

**Figure 2:**
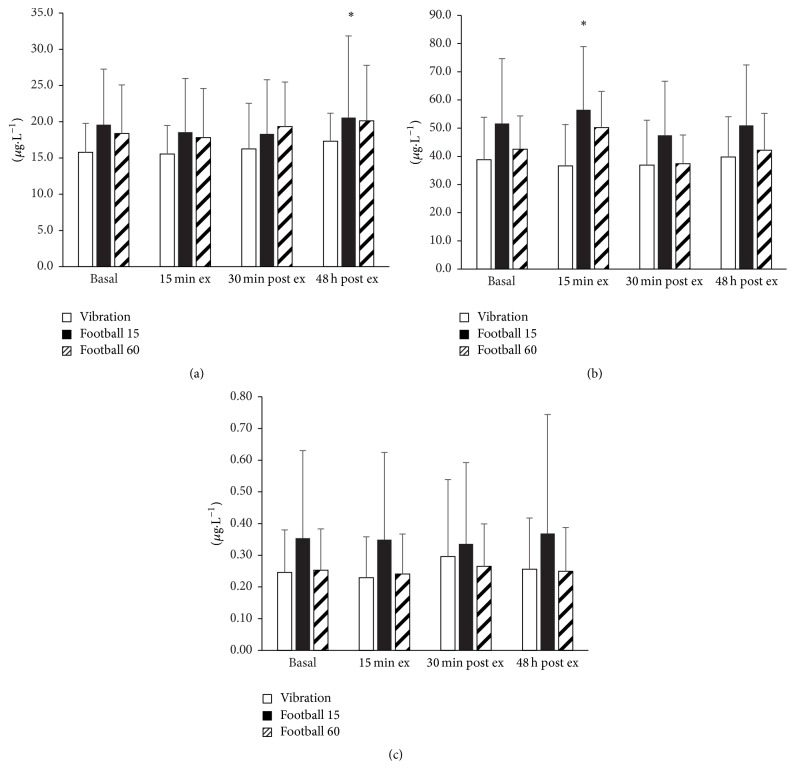
Plasma osteocalcin (a), P1NP (b), and CTX-1 (c), concentration before, during, and after the 15-minute (FG15, *n* = 13) and 60-minute (FG60, *n* = 11) football training session, and whole body vibration training (VIB, *n* = 17). ^*∗*^Significantly different from 0 min (*P* < 0.05).

There was a significant increase in plasma osteocalcin concentration in response to exercise (*P* < 0.001), which peaked 48 h after exercise (Figure 2(a)). This response was similar across all conditions (condition by time interaction effect, *P* > 0.05), increasing by 1.5 ± 2.0, 1.5 ± 3.7, and 1.7 ± 3.0 *μ*g·L^−1^ 48 h after exercise compared to basal values, respectively, for VIB, FG15, and FG60, respectively. In FG60, osteocalcin concentration did not differ between 13.5 (18.4 ± 6.2 *μ*g·L^−1^) and 54 (20.5 ± 6.4 *μ*g·L^−1^) minutes of exercise. In contrast, the exercise-induced change in plasma concentration of the bone formation marker P1NP (*P* < 0.001) was different depending upon the condition (condition by time interaction effect: *P* < 0.001, Figure 2(b)). The P1NP concentration peaked relative to baseline after 13.5 min exercise in the FG15 (14.3 ± 16.7%, *P* < 0.05) and FG60 (18.2 ± 11.1%, *P* < 0.05) groups but PINP concentration declined after 13.5 min whole body vibration exercise (−5.4 ± 7.5%, *P* > 0.05). P1NP concentration did not differ between 13.5 (48.9 ± 12.8 *μ*g·L^−1^) and 54 (48.1 ± 10.7 *μ*g·L^−1^) min of exercise in the FG60 group. There was no effect of exercise on the marker of bone resorption CTX-1, nor was there any variation in the time effect between conditions (Figure 2(c)).

## 4. Discussion

The main finding of this study was that short and longer duration small sided football session provided osteogenic, muscular, and metabolic stimuli that may contribute to the positive effects of chronic training on musculoskeletal health, with less pronounced change induced by low magnitude vibration exercise. More specifically, 48 h after a single bout of exercise, plasma osteocalcin concentration was increased by ~10% in all protocols, whereas P1NP increased by ~15% after just 13.5 min of small sided football training in both FG15 and FG60 groups. This suggests that the observed beneficial effects of chronic SSFT and VIB training programmes on BMD [11, 13, 18, 28, 29] may to some extent be attributable to repeated stimulation of osteoblast activity after each training session.

Interestingly, exercise duration did not influence the magnitude of bone marker responses, with no significant differences between FG15 and FG60 in any marker. This is despite the far greater total distance covered, and number of accelerations and decelerations completed in the FG60 versus FG15 group, and this perhaps suggests that the mechanical loading provided by 13.5 min of SSFT was sufficient to maximise signalling through the mechanotransduction pathways and stimulate osteoblast activity. Indeed exhaustive running has been shown to cause sustained increases in bone resorption markers for up to four days after exercise [30, 31] and high intensity jumping exercise until exhaustion resulted in elevated P1NP 24 h and CTX for 48 h after exercise [32]. It may therefore be advisable to adopt short sharp bursts of activity and mechanical loading such as provided by FG15 to maximise osteogenic effects.

There was a different pattern of response in plasma P1NP and osteocalcin concentrations, both in terms of time course and responsiveness to the different interventions. Osteocalcin concentration peaked at 48 h and was significantly elevated in all conditions, whereas PINP peaked after 13.5 min of SSFT but was not affected by VIB. This discrepancy most likely relates to the different processes reflected by the two bone formation markers [33]. Osteocalcin (OC) is the main noncollagen protein of bone matrix, which is primarily synthesised by osteoblasts and secreted and incorporated into the skeletal matrix. Approximately 10–30% OC is released into the circulation so plasma concentration is suggested to reflect osteoblast activity and hence bone formation [34]. In contrast to the plasma OC response, plasma P1NP concentration was elevated immediately but not 48 h after 13.5 min SSFT. P1NP is indicative of type 1 collagen synthesis, which is produced by osteoblasts [35] as well as muscle [35] and tendon [36]. Pingel et al. (2012) [37] recently employed the microdialysis technique to demonstrate increased release of P1NP from muscle but not tendon 24 h after exercise. It seems unlikely that the observed increase in plasma P1NP immediately after exercise could be due to increased muscle collagen synthesis since this is suppressed during exercise, and therefore bone seems the most likely source. We cannot exclude the possibility that exercise-induced changes in plasma volume during SSFT may contribute to the short term increases in P1NP, although the absence of increase in plasma OC concentration at the end of exercise in SSFT may argue against this. Menstrual cycle phase of participants was not controlled in this study and it is likely that this may contribute to the high degree of variation in response to exercise across participants. However, despite this limitation, differences across groups in the response to exercise were observed.

In several recent investigations small sided recreational football training has been demonstrated to cause broad spectrum adaptation in health profile [38] including enhanced bone health in premenopausal women [10, 11, 28, 39, 40]. If the acute increases in bone formation markers observed in the present study are replicated in subsequent exercise bouts, they may contribute to chronic adaptations to repeated bouts of exercise. For example football training for 15 weeks with three weekly 60-min sessions resulted in marked elevations in plasma markers of bone formation and resorption in sedentary middle-aged women [11], whereas this osteogenic response failed to occur for participants involved in continuous or interval swimming and for the inactive controls. After 15 weeks, the football training resulted in significant increases in leg bone mineral content (BMC) and femur BMD in comparison to the control group [11]. In this specific study [11], total lower extremity BMC was elevated by ~30 g, which is comparable to previous reports in men [41] and premenopausal women [10, 28]. Football training (60 min) twice per week for 16 weeks produced significant osteogenic effects and a large improvement in bone health for previously untrained female participants [39]. The acute responses observed in the present study demonstrate the potential of small sided football training to provide an effective osteogenic stimulus even after only short duration (13.5 min) exposure.

Chronic whole body vibration training has been shown to reduce markers of bone resorption [20, 21] and increase markers of bone formation [22] in a variety of populations. In the present study, there was a small increase in osteocalcin (~10%) 24 h after WBV and no change in bone resorption markers. It is not possible to elucidate whether this discrepancy relates to the lower magnitude side alternating vibration (0.9 G), different population (young women versus older mixed sex group), or the acute nature of the stimulus. To our knowledge only two studies have examined the acute effects of vibration training on bone turnover markers in adults but in both cases, VIB was performed prior to resistance training session, and therefore only the additive effects could be assessed. In both male [22] and female [23] participants, the addition of VIB training resulted in significant reductions in CTX concentration after the subsequent resistance training, but rather surprisingly bone resorption (serum Trap5b concentration) was significantly increased after VIB and resistance training in female participants. Bone formation (bone ALP) was not affected in either study, which is in contrast to the present study where VIB training alone did not alter bone resorption (CTX) but bone formation markers OC (all conditions) and P1NP (FG15 and FG60) were favorably affected. This discrepancy may relate to the time course since samples were only taken up to 30 min after training by Bemben et al. [23] and Sherk et al. [24], whereas the OC increase in the present study was observed 48 h postexercise.

There are differences in both the mechanical loading and the metabolic and hormonal responses to the SSFT versus VIB, which presumably underlie the discrepancy in the response of bone turnover markers between exercise modes. During VIB, participants were exposed to constant 0.1 G acceleration for three minutes and further seven min of 0.6 G acceleration during different static or dynamic exercises. In contrast during SSFT, participants were exposed to large numbers of low (0.1 G) and higher magnitude accelerations and decelerations that were less predictable in terms of both magnitude and direction. In 2v2 to 4v4 football session for untrained women, there are 3-4 fast runs and sideways-backwards runs per minute of play along with 3-4 so-called specific intense actions including dribbles, shots, and turns [25, 26]. These findings are supported by the results in the present study with a high number of decelerations and accelerations in both FG15 and FG60. It has been suggested that variations in strain magnitude and direction are important characteristics for the osteogenic potential of exercise [41]. In addition to these mechanical differences, blood metabolites were not affected by VIB in contrast to SSFT, where there was evidence of increased hepatic glucose output (increased blood glucose concentration), nonoxidative glycolysis (increased blood lactate concentration), altered purine nucleotide and amino acid metabolism (increased ammonia), and postexercise in particular increased lipolysis (increased FFA concentration). Interestingly, despite the shorter duration, FG15 induced similar magnitude metabolic perturbations to FG60 and similar responses in the bone turnover markers, despite the larger number, although not larger magnitude, accelerations, and decelerations in FG60. This might suggest that both FG15 and FG60 exceeded the mechanical load threshold necessary to induce changes in bone turnover or that metabolic and hormonal changes are important contributors to the acute bone turnover response to exercise. However, further studies are needed to elucidate how important the exercise duration is for the long-term bone adaptations to football training as a recent study revealed a significant positive correlation between the number of decelerations/accelerations during football training and the 12-wk increase in leg bone mass for elderly men [42].

In summary, as little as 13.5 min of small sided football altered metabolism induced muscle damage as well as exerting favorable effects on the markers of bone formation, osteocalcin, and P1NP, for up to 48 h after exercise. In contrast, the same duration of whole body vibration exercise elevated osteocalcin but not P1NP and there was not any evidence of metabolic effects or muscle damage. These acute responses may contribute to musculoskeletal health in a variety of populations after chronic small sided football.

## Figures and Tables

**Table 1:** Participant characteristics data are mean ± SD and FG60 (*n* = 18), FG15 (*n* = 18) and VIB (*n* = 20).

Parameter	FG60	FG15	VIB
Age (years)	35.7 ± 5.7	39.3 ± 5.7	39.1 ± 4.0
Height (m)	1.63 ± 0.06	1.65 ± 0.06	1.68 ± 0.05
Weight (kg)	71.9 ± 11.3	66.7 ± 10.7	74.9 ± 15.6
BMI (kg·m^−2^)	26.9 ± 4.1	24.6 ± 3.6	26.6 ± 6.0
Systolic blood pressure (mm Hg)	119 ± 12	122 ± 17	124 ± 14
Diastolic blood pressure (mm Hg)	81 ± 9	80 ± 12	79 ± 9
Calcium intake (mg·d^−1^)	1182 ± 449	1691 ± 987	1418 ± 509
Total BMD (g·cm^−2^)	1.169 ± 0.057	1.154 ± 0.080	1.203 ± 0.082
Total fat mass (kg)	29.3 ± 9.0	24.4 ± 7.4	28.7 ± 11.1
Total lean mass (kg)	39.2 ± 4.0	38.2 ± 4.2	41.8 ± 6.1

**Table 2:** Population average movement profile of the football training; data are mean ± SD and *n* = 16 FG60 and *n* = 18 FG15; *∗* indicates significant difference from FG15 and FG60 1st 15 min.

Parameter	FG15	FG60 1st 15 min	FG60 total
High intensity running (m)	163 ± 77	153 ± 58	548 ± 183^*∗*^
Total distance (m)	920 ± 178	963 ± 87	3719 ± 387^*∗*^
Accelerations (number)	80 ± 22	84 ± 23	324 ± 82^*∗*^
Decelerations (number)	123 ± 40	130 ± 39	428 ± 107^*∗*^
Mean heart rate (bpm)	156 ± 14	160 ± 19	160 ± 20
